# Epidermal growth factor receptor regulates fibrinolytic pathway elements in cervical cancer: functional and prognostic implications

**DOI:** 10.1590/1414-431X202010754

**Published:** 2021-04-19

**Authors:** F.G. Gomes, V.H. Almeida, K. Martins-Cardoso, M.M.D.C. Martins-Dinis, A.M.R. Rondon, A.C. de Melo, T.M. Tilli, R.Q. Monteiro

**Affiliations:** 1 Instituto de Bioquímica Médica Leopoldo de Meis, Universidade Federal do Rio de Janeiro, Rio de Janeiro, RJ, Brasil; 2 Instituto Nacional de Ciência e Tecnologia de Biologia Estrutural e Bioimagem, Universidade Federal do Rio de Janeiro, Rio de Janeiro, RJ, Brasil; 3 Clinical Research Division, Instituto Nacional de Câncer, Rio de Janeiro, RJ, Brasil; 4 Plataforma de Oncologia Translacional, Centro de Desenvolvimento Tecnológico em Saúde, Fundação Oswaldo Cruz, Rio de Janeiro, RJ, Brasil

**Keywords:** Cervical cancer, Epidermal growth factor receptor, Urokinase-type plasminogen activator, Thrombomodulin, Fibrinolytic system

## Abstract

Epidermal growth factor receptor (EGFR) signaling and components of the fibrinolytic system, including urokinase-type plasminogen activator (uPA) and thrombomodulin (TM), have been implicated in tumor progression. In the present study, we employed cBioPortal platform (http://www.cbioportal.org/), cancer cell lines, and an *in vivo* model of immunocompromised mice to evaluate a possible cooperation between EGFR signaling, uPA, and TM expression/function in the context of cervical cancer. cBioPortal analysis revealed that EGFR, uPA, and TM are positively correlated in tumor samples of cervical cancer patients, showing a negative prognostic impact. Aggressive human cervical cancer cells (CASKI) presented higher gene expression levels of EGFR, uPA, and TM compared to its less aggressive counterpart (C-33A cells). EGFR induces uPA expression in CASKI cells through both PI3K-Akt and MEK1/2-ERK1/2 downstream effectors, whereas TM expression induced by EGFR was dependent on PI3K/Akt signaling alone. uPA induced cell-morphology modifications and cell migration in an EGFR-dependent and -independent manner, respectively. Finally, treatment with cetuximab reduced *in vivo* CASKI xenografted-tumor growth in nude mice, and decreased intratumoral uPA expression, while TM expression was unaltered. In conclusion, we showed that EGFR signaling regulated expression of the fibrinolytic system component uPA in both *in vitro* and *in vivo* settings, while uPA also participated in cell-morphology modifications and migration in a human cervical cancer model.

## Introduction

Cervical cancer is the fourth most incident type of cancer among women, with 570,000 new estimated cases in 2018 (1), a vast majority of which occurring in developing countries. Cervical cancer is the third most common type of malignancy among Brazilian women, with a high mortality rate (2). The major risk factor for cervical cancer is human papillomavirus (HPV) infection (3).

The epidermal growth factor receptor (EGFR) has been associated with cervical cancer progression (4,5). EGFR is well described concerning its involvement in several processes such as proliferation and resistance to chemotherapy. Furthermore, EGFR expression has been correlated with poor survival among cervical cancer patients (6). HPV infection was shown to increase the amount of EGF receptors exposed on the cell membrane (by inhibiting EGFR degradation) (7) and enhance the expression of intracellular signal transducers commonly associated with EGFR signaling, such as phosphatidylinositol-3 kinase (PI3K) and extracellular signal-regulated kinase (ERK) (8). Different clinical trials have been performed employing anti-EGFR therapy for cervical cancer patients, showing ambiguous results: either with no beneficial outcomes (9), treatment-associated side effects (10), or an approximate 10% increase in overall survival (11).

A different group of receptors and secreted proteins that have been increasingly shown to participate in cancer invasion and metastasis is the fibrinolytic system. The fibrinolytic system is classically associated with the degradation of fibrin clots in the blood. However, the study performed by Malone and coworkers showed that cancer tissues from metastatic foci had increased fibrinolytic activity compared to primary non-metastatic tumor samples (12). The generation of plasmin, a key effector in the fibrinolytic system within the cancer microenvironment, has been demonstrated to be involved in extracellular matrix degradation, pro-ligand and pro-metalloprotease cleavage, and tumor cell invasion in different models (13–15). Plasmin is generated by cleavage of the circulating inactive plasminogen by plasminogen activators, such as urokinase-type plasminogen activator (uPA), which is the most relevant plasminogen activator within solid tissues. Furthermore, plasminogen can bind to different receptors on the cell surface, such as thrombomodulin (TM), annexin A2 (ANXA2), and S100 calcium-binding protein A10 (S100A10) (16,17). High expressions of uPA and uPA receptor (uPAR) have already been shown to correlate with cancer metastasis and worse patient prognosis (18).

EGFR signaling has been directly linked with components of the fibrinolytic system, for example, in breast cancer and glioblastoma. Jo et al. (19) showed that EGFR signaling was inhibited in breast cancer cells by silencing or inhibiting uPAR. Furthermore, in glioblastoma, EGFR was shown to induce uPA expression by proto-oncogene tyrosine-protein kinase Src (c-Src) and ERK signaling (20). A possible compensatory mechanism may also take place in glioblastoma where there is an increased expression of both uPA and uPAR in response to EGFR inhibition, favoring resistance to EGFR-targeted therapy (21,22).

It remains unanswered, however, if EGFR signaling is related somehow to components of the fibrinolytic system in cervical cancer. In the present work, we observed a positive expression correlation between EGFR and several fibrinolytic system elements, including uPA and TM on human cervical tumor samples, with prognostic significance. We also observed that EGFR signaling positively regulated expression of both uPA and TM in an aggressive cervical cancer cell line, and that uPA expression led to cell morphology alterations and increased migration. Finally, *in vivo*, we observed that treatment with an anti-EGFR antibody, cetuximab injected subcutaneously, reduced tumor growth, a process that was accompanied by a decreased intra-tumoral expression of uPA.

## Material and Methods

### Expression-correlation analysis

Transcriptome data from 302 patients were collected from the “TCGA, Firehouse Legacy” cohort on the platform cBioPortal (http://www.cbioportal.org/) (23,24) between May 22, 2020 and May 23, 2020. This cohort had 304 patients (as of May 23, 2020) two of which had two samples taken (totaling 306 samples from 304 patients). These two patients were excluded from the analysis, therefore the total number of samples/patients shown in the present work is 302. mRNA expression (RNA Seq V2 RSEM) in FPKM (frames per kilobase per million) data was downloaded, plotted using Graphpad Prism^TM^ (USA), and analyzed by non-parametric Spearman correlation.

### Overall survival analysis

From the cBioPortal platform, we established a cut-off of 1.5 standard deviations above the median of expression to separate groups of “overexpression” and “cases without alteration” of the cohort “TCGA, Firehouse Legacy”, from the 302 patients. After the establishment of the gene expression cut-off, we analyzed overall survival at the “survival” window on cBioPortal, extracted the data, and assembled the graphs with Graphpad^TM^. Mantel-Cox test was used for statistical analysis.

### Cell lines

Human cervical cancer cells CASKI (more aggressive) and C-33A (less aggressive) were cultivated in RPMI medium supplemented with 10% fetal bovine serum (FBS), and maintained in 37°C, 5% CO_2_. CASKI cell line was originated from an intestinal metastasis of an epidermoid cervical carcinoma and was infected with multiple copies of HPV-16 (ATCC, CRL-1550™). C-33A cells originated from a primary epidermoid cervical carcinoma, uninfected by HPV (ATCC, HTB-31™).

### Real-time PCR

Briefly, C-33A or CASKI were seeded (4×10^5^ cells/well) onto 6-well plates, in RPMI medium supplemented with 10% FBS. The next day, the wells were washed with phosphate-buffered saline (PBS) and cells were starved (FBS-free medium) for 16 h. After starving, cells were treated with anti-EGFR antibody (cetuximab, Merck, Germany, 100 µg/mL), PI3K inhibitor (LY294002, Sigma-Aldrich, USA, 25 µM), or MEK inhibitor (PD98059, Sigma-Aldrich, 50 µM), for 1h. Then, cells were treated with EGF (50 ng/mL) for 1.5 h or 3 h, 37°C, 5% CO_2_. The wells were washed with PBS and 0.5-1 mL Trizol (Sigma-Aldrich) was added to each well. The extracts were transferred into microcentrifuge tubes and stored at -20°C, for posterior RNA isolation. From each sample, 1 µg of total RNA was reverse transcribed to cDNA. Next, quantitative PCR was performed on cDNA aliquots.

For TM real-time PCR, 5 µL master mix (Thermo Fisher Scientific, USA), 0.5 µL TaqMan probe for human TM (Hs00264920_s1) or human GAPDH (reference gene, 4326317E), 2 µL injection water, and 2.5 µL diluted cDNA (1:20 v/v) were added, totaling 10 µL per well of a 96-well PCR plate. Quantitative PCR was performed with a StepOnePlus™ real-time PCR system (Thermo Fisher Scientific). The cycling conditions were: 95°C (20 s), followed by 50 cycles of 95°C (1 s) and 60°C (20 s).

For uPA real-time PCR, 7.5 µL SYBR green mix, 0.6 µL forward primer (10 µM), 0.6 µL reverse primer (10 µM) (both primers purchased from Thermo Fisher Scientific), 4.8 µL injection water, and 1.5 µL cDNA (diluted 1:20 v/v) were added, totaling 15 µL per well of a 96-well PCR plate. The cycling conditions were: 95°C (10 min), followed by 45 cycles of 95°C (15 s) and 60°C (1 min). Finally, the melting curve was 95°C (15 s), 60°C (1 min), and 95°C (15 s). In this case, 18S was used as a reference gene (25) (Table 1). The (1 + efficiency)^−ΔΔCT^ method was used to analyze the fold increase.

**Table 1:** Sequence and product size for the primers used on SYBR green real-time PCR protocol.

Gene name	Primers	Product size
uPA	F: GTCACCTACGTGTGTGGAGG	147 base pairs
	R: CTTCATCTCCCCTTGCGTGT	
18S	F: AACCCGTTGAACCCCATT	149 base pairs (ref. 25)
	R: CCATCCAATCGGTAGTAGCG	

### Plasmin enzymatic activity assay

CASKI or C-33A cells were seeded (3×10^4^/well) onto 96-well plates in RPMI medium supplemented with 10% FBS. When necessary, 100 µg/mL cetuximab was added to the wells after seeding and incubated for 30 min, 37°C, 5% CO_2_. Then, EGF was added to the wells of interest at 10, 50, or 100 ng/mL and the plates were incubated for 18 h at 37°C, 5% CO_2_. The wells were washed twice with FBS/phenol red-free medium and uPA inhibitor (BC-11 hydrobromide, Abcam, USA) was added at a final concentration of 50 µM for approximately 15 min at room temperature. After BC-11 incubation, lys-plasminogen was added at 0.5 µM, and 250 µM of plasmin-chromogenic substrate S2251 (Diapharma, USA) was immediately pipetted to the wells. Plates were analyzed on a Spectramax 190 plate reader (Molecular Devices, USA) for 5 h, at 37°C, during which 405 nm reads were performed at 4 min intervals. All experiments were performed in duplicate.

### Flow cytometry

Flow cytometry assays were performed using an anti-TM antibody conjugated with the APC (allophycocyanin) fluorophore, which has peak excitation at 645 nm and emission at 660 nm. Briefly, 5×10^5^ cells were incubated with 2.5 µL anti-TM antibody in 100 µL final volume, 12.5 µg/mL final concentration (BioLegend, clone M80, USA), for 30 min, on ice. The cells were then washed with PBS and resuspended on 400 µL of FACs buffer (PBS + 5% FBS). Basal TM expression of C-33A and CASKI cells was evaluated on a FACSVerse^TM^ machine (BD Biosciences, USA). TM-protein expression was also evaluated after stimuli with 50 ng/mL EGF +/- cetuximab, for 6 h. The experiment was performed on an Accuri^TM^ (BD Biosciences) flow cytometer, evaluating the percentage of positive events and mean fluorescence intensity (MFI).

### Cell morphology and migration

CASKI cells (5×10^5^/well) were seeded onto 6-well plates. After 24 h, cells were starved in FBS-free media for 10 h and treated with or without 0.5 µM glu-plasminogen, 100 µg/mL cetuximab, and/or 50 µM BC-11 for approximately 16 h at 37°C, 5% CO_2_. Plates were photographed with a Qimaging camera (Q35443, CE, Canada), at 10× magnification. Immediately after the photographs, cells were harvested for the Boyden chamber migration assay, which was performed using 8-μm pore polycarbonate membranes (Costar, USA). The lower compartments were filled with RPMI media containing 5% FBS and 3×10^4^ treated cells were seeded in 50 μL serum-free media to the upper chambers. After 20 h of incubation, at 37°C with 5% CO_2_, the non-migrated cells in the upper chambers were removed and the membranes were fixed and stained using the fast Panoptic staining kit (Laborclin, Brazil). Ten image fields were evaluated for each condition. The mean number of cells per field was calculated for each condition (of each experiment) and plotted.

### *In vivo* assays

For *in vivo* assays, 1×10^7^ CASKI cells were subcutaneously injected into both flanks of immunocompromised Balb/c nude female mice. One week later, when tumors became palpable, the size of tumors was calculated using the following formula: V = D × d^2^ / 2, where V: tumor volume (mm^3^); D: larger tumor mass diameter (mm); d: smaller tumor diameter (mm).

The measurements were made using a caliper. Day 0 was defined as the day when the cell injections were made, and then tumors were measured on days 7, 9, 12, 14, and 15. Injected animals were separated into two groups: 1) Control group: treated intraperitoneally with 200 µL PBS (4 animals); and 2) Intervention group: treated intraperitoneally with 200 µL cetuximab (1 mg/animal, 3 animals). Both tumors in each animal were analyzed as an independent experimental unit. Treatments were performed on the same day of size measurements, except for day 15. On day 15, the animals were anesthetized with ketamine/xylazine and euthanized by cervical displacement. Immediately after euthanasia, tumors were excised using scissors and scalpel and weighed on a precision scale. Finally, tumors were cut into smaller pieces with a scissor, inserted into 1.5 mL Eppendorf tubes containing Trizol, and stored at -20°C. Samples were then macerated using a Turrax homogenizer (Daigger Scientific, USA), which was washed with DEPC water, Trizol, 75% ethanol, and DEPC water, in this order, between samples. Samples were then further processed for real-time PCR. The use of animals in this work was approved by the Commission of Ethics in Animal Use of the Federal University of Rio de Janeiro, registered at the National Control Council of Animal Experimentation under the process number 01200.001568/2013-87, protocol 102/16.

### Data analysis and presentation

When comparing two groups, one-sample *t*-test (when one of the group's data had been normalized to “1”) or two-sample unpaired two-tailed *t*-test was employed. For multiple comparisons, one-way ANOVA with Tukey's post-test was used. All statistical analyses were done with Graphpad Prism^TM^. When applicable, data are reported as means±SD (with the exception of Figure 6A, which reports means±SE). Experimental units for *in vitro* assays were considered to be the mean result of each experiment, performed on different cell-culture passages.

## Results

### EGFR, uPA, and TM expressions were positively correlated in samples from human cervical cancer tumors, which correlated with poor overall survival

**Figure 1:**
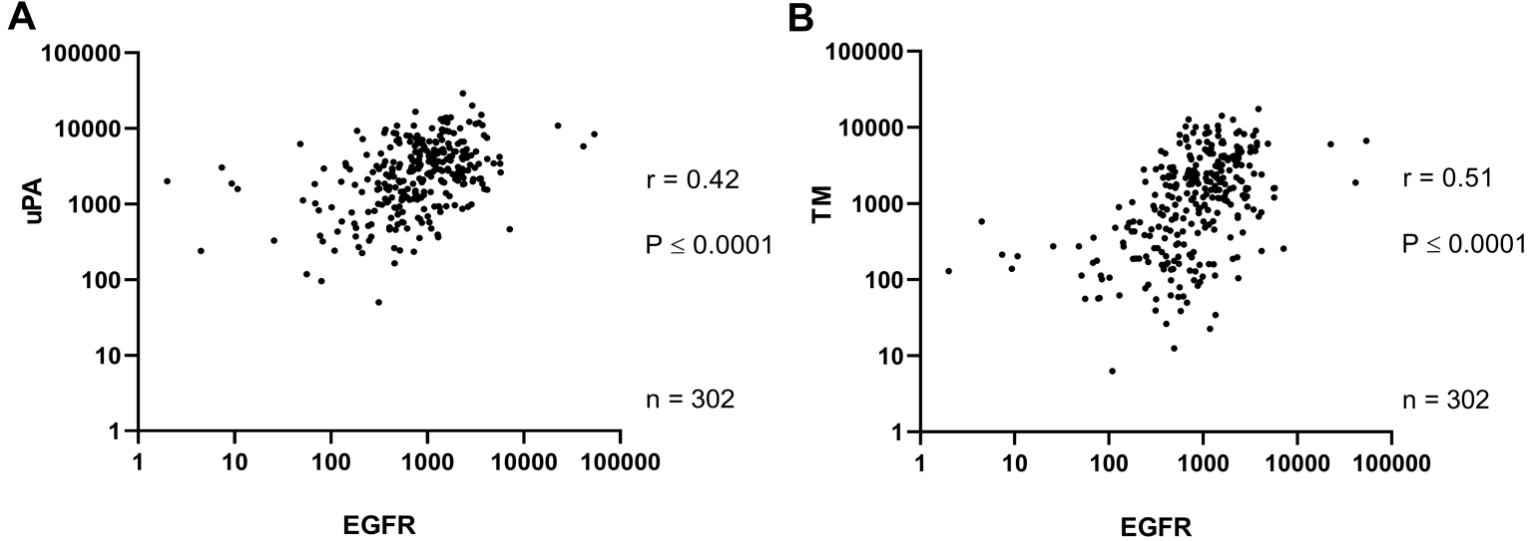
Epidermal growth factor receptor (EGFR), urokinase-type plasminogen activator (uPA), and thrombomodulin (TM) expressions have a positive correlation in samples from human cervical tumors. Data are reported as FPKM (frames per kilobase per million). Spearman correlation test was used for statistical analysis.

**Figure 2:**
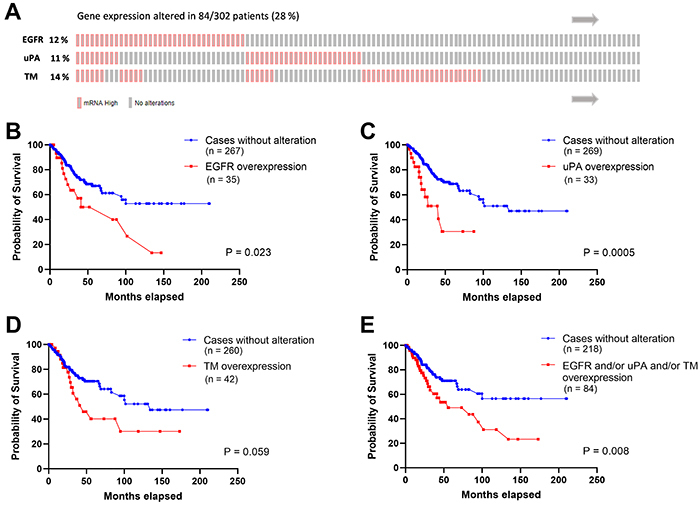
Overall survival analysis of cervical cancer patients concerning epidermal growth factor receptor (EGFR), urokinase-type plasminogen activator (uPA), and thrombomodulin (TM) tumor overexpression. **A**, Map of the alterations in the EGFR, TM, and uPA gene expression in tumor samples of 302 cervical cancer patients. Patients who exhibited overexpression of these genes in the tumor are indicated in red. This map was generated through cBioPortal, which is based on the clinical and molecular information provided by TCGA. Overall survival analysis of cervical cancer patients with gene expression of 1.5 standard deviations above the median for EGFR (**B**), uPA (**C**), TM (**D**), or any of the three genes, in an isolated or concomitant manner (**E**). Survival curve comparison was done through the Log-rank (Mantel-Cox) statistical test.

A positive correlation between EGFR x uPA expressions (r=0.42, P<0.0001, Figure 1A) and EGFR x TM expressions (r=0.51, P<0.0001, Figure 1B) was observed in cervical cancer samples. In the criteria established in the current study, the mRNA upregulation of EGFR, uPA, and TM was found in 12, 11, and 14% of the cases, respectively (Figure 2A). Furthermore, cervical cancer patients with increased expression of EGFR (P=0.023, Figure 2B), uPA (P=0.0005, Figure 2C), or TM (P=0.059, Figure 2D) had decreased overall survival compared to the patients with normal levels. The effect of the upregulation in one, two, or all three genes in this group of patients (n=84) was also significantly associated with poor overall survival (P=0.008, Figure 2E). A positive correlation between EGFR expression and other components of the fibrinolytic system (Table S1) was also observed, although the increased expression of each gene showed no correlation with alterations of overall survival of the cervical cancer patients (Supplementary Figure S1).

### CASKI cells expressed higher levels of EGFR, uPA, and TM compared to C-33A cells

**Figure 3:**
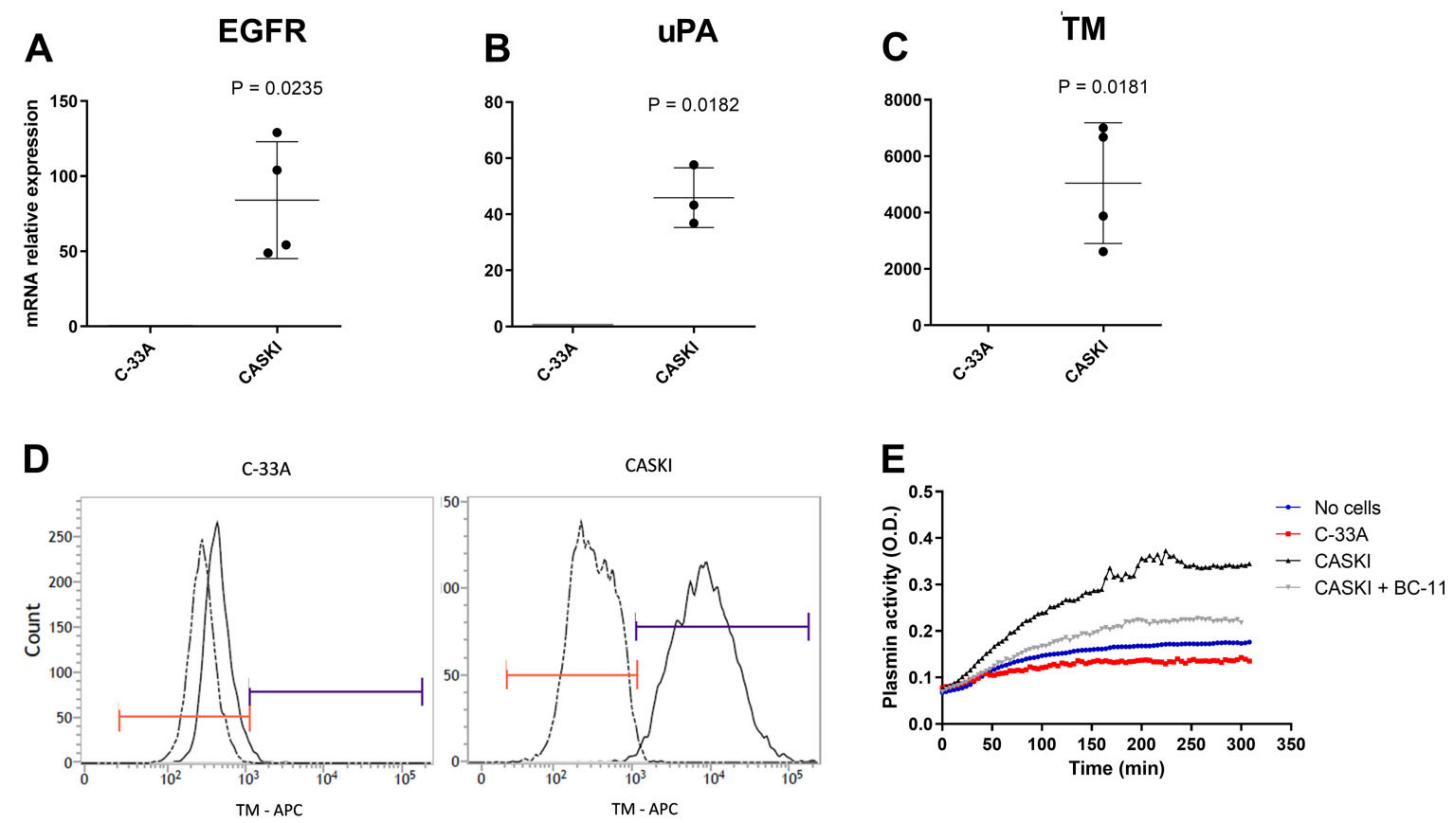
CASKI cells express higher levels of epidermal growth factor receptor (EGFR), urokinase-type plasminogen activator (uPA), and thrombomodulin (TM) compared to C-33A cells (**A**-**C**). Data are reported as means±SD of 3-4 independent experiments (unpaired *t*-test). **D**, Representative histograms of the flow cytometry for TM of C-33A and CASKI cells, analyzing the percentage of positive events. **E**, Representative graph of the plasmin enzymatic activity assay of C-33A and CASKI cells (+/- uPA inhibitor, BC-11, 50 µM) followed by 0.5 µM lys-plasminogen and 250 µM chromogenic substrate S2251, analyzed over 5 h with 4 min between each read at 405 nm.

The more aggressive CASKI cells expressed higher mRNA levels of the EGFR, uPA, and TM (Figure 3A-C) genes compared to C-33A. On a protein level, C-33A cells were negative for TM, as observed by flow cytometry, whereas CASKI cells were mostly positive (Figure 3D). C-33A cells also did not generate active plasmin from added plasminogen (reaction catalyzed by cell-produced plasminogen activators, such as uPA) while, in contrast, CASKI cells had an uPA-dependent generation of active plasmin (Figure 3E). Previously published data from our group shows that CASKI cells have higher protein levels of EGFR compared to C-33A cells (26).

### EGFR signaling upregulated uPA and TM expression in CASKI cells

**Figure 4:**
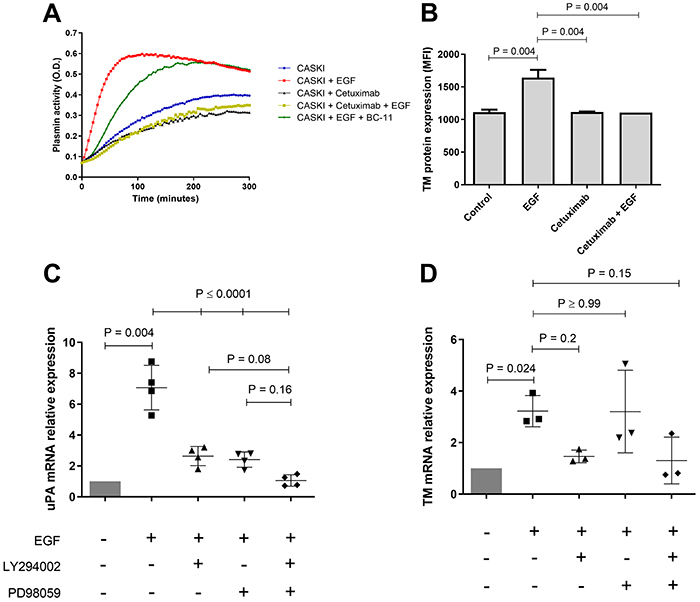
Epidermal growth factor receptor (EGFR) signaling upregulates urokinase-type plasminogen activator (uPA) and thrombomodulin (TM) expression in CASKI cells. **A**, Representative graph of the plasmin enzymatic activity assay performed with CASKI cells treated overnight with epidermal growth factor (EGF) alone, cetuximab alone, EGF 30 min after cetuximab treatment, or EGF overnight plus BC-11 15 min before starting measurements. **B**, Flow cytometry staining for TM in CASKI cells treated with EGF, cetuximab, or the combination of both. Data are reported as mean fluorescence intensity±SD. One-way ANOVA with Tukey's post-test was used for statistical analysis. Real-time PCR for uPA (**C**) or TM (**D**) in CASKI cells treated with EGF +/- LY294002, +/- PD98059. A one-sample *t*-test was performed for comparisons to the normalization control “CASKI”, and one-way ANOVA with Tukey's post-test was performed for the remaining comparisons. In all cases, LY294002 and PD98059 were added to the cells 1 h before EGF addition, which was then done for 15 min (western blot) or 1.5 h (real-time PCR). Final concentrations were: 50 ng/mL EGF, 25 µM LY294002, and 50 µM PD98059.

On a protein level, the addition of the anti-EGFR monoclonal antibody cetuximab reduced basal plasmin generation, as well as reverted the EGF-mediated increase of plasmin activity (Figure 4A) in CASKI cells. EGF addition to C-33A cells did not alter uPA expression or plasmin generation (Supplementary Figure S2). EGF-induced plasmin generation in CASKI cells was promoted by uPA (Figure 4A). TM protein expression increased with EGF addition to CASKI cells, and such effect was reverted by cetuximab (Figure 4B, Supplementary Figure S3).

In order to evaluate whether EGFR signaling had a direct impact on uPA and TM expression, CASKI cells were incubated with EGF with or without inhibitors of the downstream EGFR-signaling branches PI3K-Akt (LY294002) and MEK1/2-ERK1/2 (PD98059) (Figure 4C and D). EGF-induced uPA expression was significantly diminished in CASKI cells by both inhibitors (Figure 4C), whereas EGF-induced TM expression was significantly diminished by PI3K inhibition (Figure 4D).

### uPA participated in morphology-altering and migration phenomena in CASKI cells

**Figure 5:**
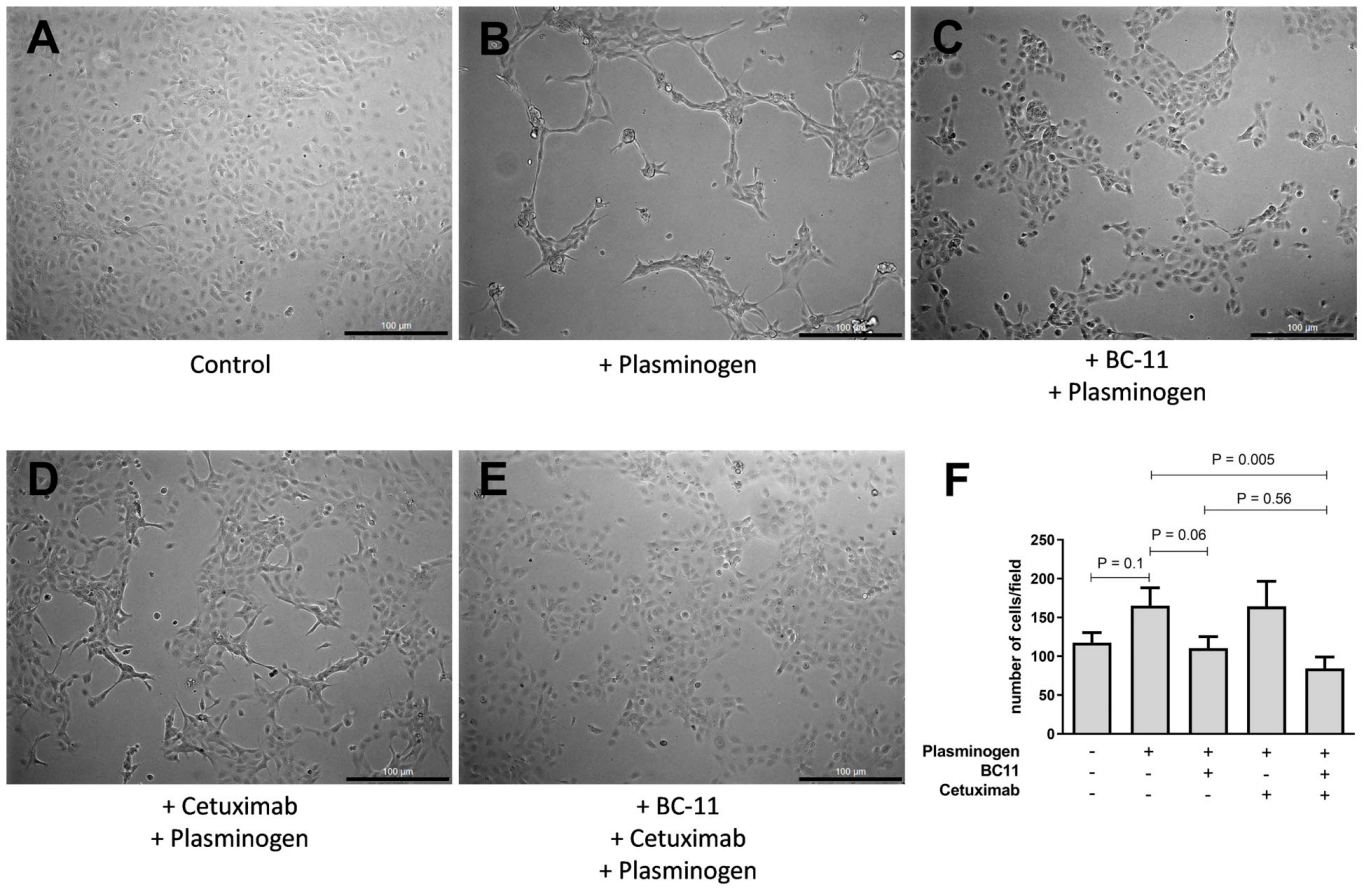
Epidermal growth factor receptor (EGFR)/urokinase-type plasminogen activator (uPA) participated in morphology-altering and migration phenomena in CASKI cells. **A**-**E**, Bright field light microscopy pictures (100× magnification, scale bar 100 μm) of CASKI cells treated for 16 h with plasminogen +/- uPA inhibitor (BC-11) or +/- anti-EGFR monoclonal antibody (cetuximab). **F**, Cell migration experiment in the Boyden chamber was performed on the cells treated the same as in the microscopy images. Final treatment concentrations: 0.5 µM glu-plasminogen, 50 µM BC-11, and 100 µg/mL cetuximab. Data are reported as means±SD of three independent experiments. One-way ANOVA with Tukey's post-test was used for statistical analysis.

To assess the possible functional impact that EGFR-dependent plasmin generation may have on CASKI cells, we evaluated the cell morphology under different treatments (Figure 5A-E). Upon plasminogen addition, cells changed their morphology into a highly connected state, assembling into “branched” structures composed of overlapping cells, as opposed to the control-monolayer cell disposition (Figure 5A and B). Both uPA and EGFR inhibition, in an isolated manner, hampered this plasminogen-induced cell-morphology alteration (Figure 5C and D) and the combination of both inhibitors seemed to reverse this effect to a state that mostly resembled the control group (Figure 5E).

Cell migration experiments were performed immediately after cell-morphology images were taken and, although plasminogen addition led to a trend of increased cell migration on a Boyden chamber model, EGFR or uPA inhibition alone did not decrease plasminogen-induced cell migration, whereas the combination of both inhibitors significantly hampered this process (Figure 5F).

### *In vivo* treatment with cetuximab decreased tumor growth/weight and intra-tumoral uPA expression

**Figure 6:**
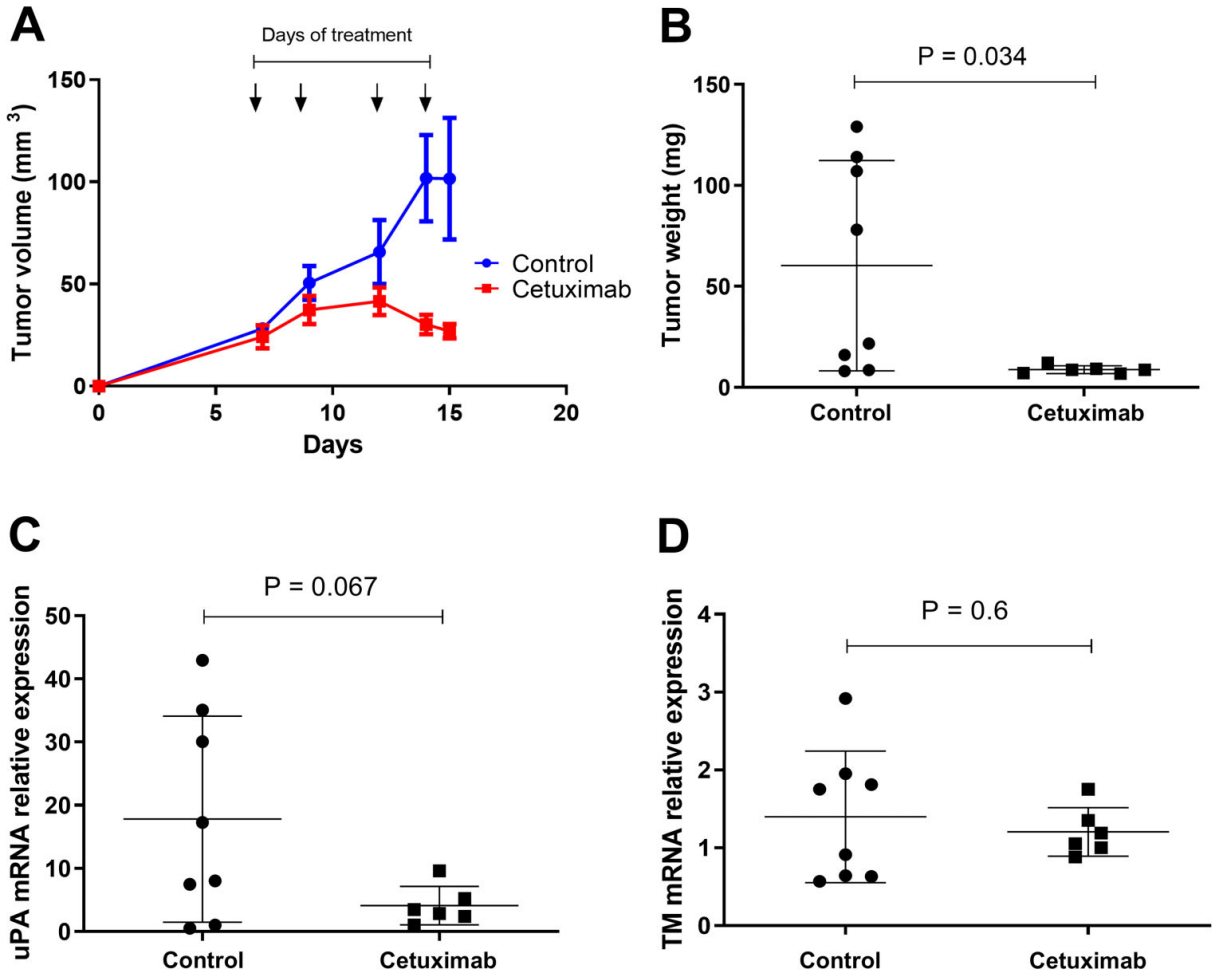
*In vivo* treatment with cetuximab decreased tumor growth/weight and intra-tumoral urokinase-type plasminogen activator (uPA) expression. **A**, Tumor growth of subcutaneously injected CASKI cells (1×10^7^) into Balb/c nude mice. The arrows indicate the time points in which PBS or 1 mg cetuximab were administered intraperitoneally in a final volume of 200 µL per animal. After euthanasia of the animals, tumors were excised, weighed (**B**), prepared for RNA extraction, and quantitative real-time PCR was performed for uPA (**C**) and thrombomodulin (TM) (**D**). Two-sample Student's *t*-test was used for statistical analysis comparing control group (n=8) *vs* cetuximab group (n=6).

Cetuximab treatment reduced *in vivo* tumor growth and weight (Figure 6A and B) and led to a trend of uPA expression reduction within the tumor tissue (Figure 6C), whereas TM expression was unaffected by *in vivo* cetuximab treatment (Figure 6D).

## Discussion

Cervical cancer is a type of malignancy that, unfortunately, still imposes a great burden on women living in low and middle income countries. Although the standard treatment consisting on surgical resection for initial disease or chemotherapy in combination with radiotherapy for locally advanced disease is of fundamental importance, there is still a great need for: 1) new therapeutic targets and 2) biomarkers for screening patients that could benefit more from certain targeted therapies. EGFR has been considered to be a receptor involved in cervical cancer progression (4,5), however, clinical trials are still not conclusive as to whether blocking EGFR is effective enough for justifying its use in the clinical setting (9–11). Furthermore, it has been described that, in other types of cancer in which anti-EGFR treatment is Food and Drug Administration-approved, it is quite common for patients to develop resistance mechanisms towards EGFR-targeted therapy (27).

In the present work, EGFR, uPA, and TM expression had a positive correlation on human cervical tumor samples, and the high expression of these genes was associated with worse patient overall survival. A meta-analysis published in 2002, with data from 8,377 breast cancer patients, showed that increased uPA levels correlated with worse disease-free and overall survival (28). Serum uPA levels in 252 breast cancer patients were positively correlated with worse progression-free and overall survival, as well as associated with an increase in plasma HER2 levels, a receptor of the EGFR family (29). In 2014, however, another study showed that plasma levels of uPA did not correlate with lymph node metastasis status, also in human breast cancer (30). In colorectal cancer, uPA levels in plasma or primary site *vs* normal mucosa did not correlate with alterations in patient survival (31). In cervical cancer, Sugimura et al. showed that uPA staining in primary tumor biopsies correlated positively with lymph node metastasis status (32). Another group in 2002, however, did not observe any prognostic impact of intra-tumoral uPA, also among cervical cancer patients (33).

It has been shown that the uPA receptor, uPAR, can transactivate EGFR (19) and that both uPA and uPAR participate in the resistance mechanisms towards anti-EGFR treatment in glioblastoma (21,22). One hypothesis that arises in light of these results is that, in cervical cancer, there may be a positive feedback loop, in which EGFR positively regulates uPA expression, and that its receptor, uPAR, may favor EGFR transactivation, as has been shown in glioblastoma (19). Furthermore, the silencing of uPA and uPAR in a pancreatic cancer model reduces *in vivo* tumor growth and angiogenesis (34). A clinical trial has been published using an uPA inhibitor called WX-671 in locally advanced pancreatic cancer, showing no notable difference between groups treated with chemotherapy alone or in combination with uPA inhibition (35). Another clinical trial using a competitive inhibitor of the binding between uPA and uPAR, called A6, showed minimal beneficial effects on patients with gynecological tumors such as the ovary, fallopian tube, and peritoneum (36). Perhaps finding signaling partners for either EGFR or uPA could be useful in optimizing therapeutic efficiency for these targets.

In the present work, we showed that EGFR, uPA, and TM levels of human cervical tumors correlated with worse overall patient survival and that uPA and TM were positively regulated by EGFR. Furthermore, uPA expression can have a direct impact on cervical cancer cell function, specifically EGFR-dependent cell morphology modifications and EGFR-independent cell migration. To the best of our knowledge, we are the first to show this interplay between EGFR signaling and components of the fibrinolytic system in cervical cancer. Therefore, we propose uPA as a potentially novel candidate to be studied in combination with EGFR in cervical cancer, either as a therapeutic target or as a biomarker for EGFR signaling and patient prognosis.

## Supplementary Material

Click here to view [pdf].
